# Surgical Handover Core Outcome Measures (SH-CORE): a protocol for the development of a core outcome set for trials in surgical handover

**DOI:** 10.1186/s13063-024-08201-x

**Published:** 2024-06-10

**Authors:** Jessica M. Ryan, Declan Devane, Anastasija Simiceva, Walter Eppich, Dara O. Kavanagh, Christine Cullen, Aisling M. Hogan, Deborah A. McNamara

**Affiliations:** 1 RCSI SIM Centre for Simulation Education and Research, 123 St. Stephen’s Green, Co. Dublin, Ireland; 2 RCSI StAR MD Programme, St. Stephen’s Green, Co. Dublin, Ireland; 3 The Bon Secours Hospital, Glasnevin Hill, Glasnevin, Co. Dublin, Ireland; 4 School of Nursing and Midwifery, Áras Moyola, University of Galway, Co. Galway, Ireland; 5 Health Research Board, Trials Methodology Research Network, Áras Moyola, University of Galway, Co. Galway, Ireland; 6 Evidence Synthesis Ireland and Cochrane Ireland, University of Galway, Co. Galway, Ireland; 7 Department of Medical Education and Collaboratory Practice Centre, The University of Melbourne, Melbourne, Australia; 8 Department of Surgical Affairs, RCSI, 121 St. Stephen’s Green, Co. Dublin, Ireland; 9 Department of Surgery, Tallaght University Hospital, Tallaght, Co. Dublin, Ireland; 10 Patient and Public Partner, Co. Dublin, Ireland; 11 Department of General Surgery, Galway University Hospital, Co. Galway, Ireland; 12 Office of the President, RCSI, 123 St. Stephen’s Green, Co. Dublin, Ireland; 13 National Clinical Programme in Surgery, RCSI, 2 Proud’s Lane, Co. Dublin, Ireland; 14 Department of Surgery, Beaumont Hospital, Beaumont, Co. Dublin, Ireland

**Keywords:** Core outcome set, Core outcome measure, Consensus method, Handover, Handoff, Delphi survey, Surgical handover, Surgical communication, Handover methodology, Information transfer, SBAR, I-PASS, Signoff, Signout

## Abstract

**Background:**

Surgical handover is associated with a significant risk of care failures. Existing research displays methodological deficiencies and little consensus on the outcomes that should be used to evaluate interventions in this area. This paper reports a protocol to develop a core outcome set (COS) to support standardisation, comparability, and evidence synthesis in future studies of surgical handover between doctors.

**Methods:**

This study adheres to the Core Outcome Measures in Effectiveness Trials (COMET) initiative guidance for COS development, including the COS-Standards for Development (COS-STAD) and Reporting (COS-STAR) recommendations. It has been registered prospectively on the COMET database and will be led by an international steering group that includes surgical healthcare professionals, researchers, and patient and public partners. An initial list of reported outcomes was generated through a systematic review of interventions to improve surgical handover (PROSPERO: CRD42022363198). Findings of a qualitative evidence synthesis of patient and public perspectives on handover will augment this list, followed by a real-time Delphi survey involving all stakeholder groups. Each Delphi participant will then be invited to take part in at least one online consensus meeting to finalise the COS.

**Ethics and dissemination:**

This study was approved by the Royal College of Surgeons in Ireland (RCSI) Research Ethics Committee (202309015, 7th November 2023). Results will be presented at surgical scientific meetings and submitted to a peer-reviewed journal. A plain English summary will be disseminated through national websites and social media. The authors aim to integrate the COS into the handover curriculum of the Irish national surgical training body and ensure it is shared internationally with other postgraduate surgical training programmes. Collaborators will be encouraged to share the findings with relevant national health service functions and national bodies.

**Discussion:**

This study will represent the first published COS for interventions to improve surgical handover, the first use of a real-time Delphi survey in a surgical context, and will support the generation of better-quality evidence to inform best practice.

**Trial registration:**

Core Outcome Measures in Effectiveness Trials (COMET) initiative 2675. http://www.comet-initiative.org/Studies/Details/2675.

## Introduction

Handover refers to the exchange of information about a patient at the time of transfer of responsibility for the patient’s care [1]. One-quarter of handovers are associated with care failures [2] and 20% of handover-related issues lead to adverse events [3], making this a vulnerable point in the patient journey. During hospitalisation for surgical conditions, care handovers happen frequently, yet standardised processes are rare. There is no universally accepted standard for surgical handover [4], and whilst multiple organisations offer general guidance [5, 6], no evidence-based guidelines exist for safe and effective surgical practice. In addition to a lack of high-quality research, there is no consensus on which outcomes are relevant and most important to stakeholders when evaluating interventions to improve surgical handover between doctors. A recent systematic review in this area [7] identified deficiencies in handover research methodology, including significant heterogeneity in the outcomes measured and reported.

A core outcome set (COS) is 'an agreed standardised set of outcomes that should be measured and reported, as a minimum, in all clinical trials in specific areas of health or healthcare’ [8]. They represent the most relevant outcomes of importance, developed through a consensus-driven approach, incorporating the views of all relevant groups of stakeholders. A COS supports standardisation of outcome measurement and trial design, thereby enhancing comparability across studies and evidence synthesis and reducing outcome-reporting bias. Additionally, incorporating the patient and public perspective in COS development ensures that outcomes of importance to patients are also captured [9].

This paper presents the protocol for the development of a COS for studies evaluating interventions to improve surgical handover between doctors. A consensus-driven process will involve surgical healthcare professionals, researchers, and patients to develop the first published COS for healthcare handover.

## Methods

This protocol has been developed and reported according to the Core Outcome Set-Standardised Protocol Items (COS-STAP) [10]. The planned study has been developed according to the Core Outcome Measures in Effectiveness Trials (COMET) initiative guidance for core outcome set development [8] and will be conducted and reported following the Core Outcome Set-Standards for Development (COS-STAD) [11] and Reporting (COS-STAR) [12] recommendations. It has been prospectively registered on the COMET database (registration no. 2675), available online. The authors are not aware of any similar published COS.

### Study summary

A multi-phase process is proposed, beginning with a systematic review of all published literature on interventions to improve any handover between surgical doctors and a separate review of qualitative literature to identify candidate patient outcomes. Stakeholder representatives will then take part in a real-time (RT) Delphi survey, followed by one or more online consensus meetings to finalise the COS.

### Steering committee

The phases of this study will be overseen by an international steering committee comprising representatives from three stakeholder groups, including healthcare professionals (HCPs) working in surgery, researchers and methodologists (including those with expertise in COS development), and patient and public partners. The steering committee will convene periodically to review progress and guide project decisions.

### Definitions

For this COS, surgical handover is defined as the exchange of information about a patient that occurs between surgical doctors at the time of transfer of responsibility for the patient’s care. This includes doctors working in any surgical specialty, at any timepoint in the patient journey. An intervention is a deliberate action or strategy implemented with the purpose of improving handover-related outcomes. Interventions can use any combination of methods but must aim to improve the structure or content of handover. Outcomes are defined as measures used to assess the effect of an intervention.

### Phase one: review of published literature and generation of outcome list

#### Systematic review

A systematic review of studies evaluating the impact of interventions targeting any handover between surgical doctors on all outcomes was carried out, including papers published from database inception up to April 2023 (PROSPERO registration number CRD42022363198). All surgical specialties were included. The full details of the search and data synthesis will be published in a separate manuscript (in press). An initial list of outcomes was developed during this process and categorised using a novel taxonomy developed by the authors.

#### Qualitative review

The views of all relevant stakeholders should be considered in the development of a COS [11]. For this study, a review of qualitative literature reporting on patient perspectives of in-hospital handover will be performed. Synthesising the views, attitudes, and experiences of patients, carers, and family members will help to identify additional outcomes, as the patient perspective was not well-represented in the interventional studies. This review will be registered prospectively on PROSPERO, the international prospective register of systematic reviews, and will be conducted and reported following the Preferred Reporting Items for Systematic Reviews and Meta-Analyses (PRISMA) guidelines [13] and the Enhancing Transparency in Reporting the Synthesis of Qualitative Research (ENTREQ) guidelines [14].

#### Generation of outcome list

Outcomes identified during the qualitative review which are not relevant to surgical handover between doctors will be excluded from the long list, the remaining outcomes will be mapped to domains within the outcome taxonomy. Outcomes from both reviews will be refined and combined where appropriate, and a long list of outcomes for the RT Delphi survey will be generated. They will be categorised according to a taxonomy of handover outcomes developed by the authors during the systematic review (in press).

### Phase two: Delphi survey

The long list of outcomes will be incorporated into a RT Delphi survey. Plain language explanations for all outcomes identified will be used where available; otherwise de novo explanations will be developed with the help of artificial intelligence. These explanations will be reviewed iteratively by patient and public partner steering committee members, with amendments made where necessary. To increase the retention of participants, a RT Delphi survey will be utilised, which has been demonstrated in a recent randomised controlled trial (RCT) to be non-inferior to the traditional multi-round Delphi survey [15].

#### Stakeholder groups

A purposive international sample of (1) surgical HCPs (surgeons, nurses, residents, physician associates, etc.), (2) former surgical patients, their carers or family members (i.e. ‘patient and public partners’), and (3) researchers with experience in surgical handover or core outcome set development will be invited to take part in the RT Delphi survey. There are no prerequisite criteria for sample size for participation in Delphi studies, and panel size usually depends on the availability of experts and resources [16]. There is evidence to suggest that a small panel of experts (*n*= 23) in a ‘well-defined knowledge area’, selected via strict inclusion criteria can produce stable results [16]. As such, the aim is to include around 40 participants in each stakeholder group with international representation. HCPs and researchers will be identified through published literature, international guidelines, social media, and conferences. Patient and public partners will be invited through multiple channels, including social media and patient support groups. All stakeholders will be invited to participate by gate keepers. Information leaflets and consent forms will be provided electronically.

#### Real-time (RT) Delphi survey

The methodology for this process has been guided by the RCT protocol published by Quirke et al. (2021) [17]. Before commencing the RT Delphi, a representative group of stakeholders (at least five from each of the three groups) will be asked to complete the survey, so that consensus information is available to subsequent RT Delphi participants. Participants will be asked to rate outcomes using a 9-point Likert scale after providing demographic information [8]. The order in which outcomes are presented to participants will be randomised, as question order may affect participant response [18]. A plain language explanation of the terms used will also be provided [19]. Once a participant has rated an item, they will be presented with results for that outcome in real-time, including their own rating, the overall rating, and the rating for each stakeholder group. Based on this result, they can choose to change or retain their initial rating. Participants will also be invited to propose additional outcomes that they feel are important via free-text response. These suggestions will be reviewed by the steering group within 1–3 days and added to the RT Delphi if suggested by two or more participants. As the level of consensus changes during this process, participants, including the initial group of respondents, will be encouraged to review the survey again. Participants can re-visit the survey to complete or modify it as they wish during the survey period.

#### Delphi consensus definition

In keeping with the guidance provided in the COMET handbook [8], outcomes will be retained after the RT Delphi if they are scored between 7 and 9 by at least 70% of participants in each stakeholder group and between 1 and 3 by less than 15%. Outcomes will be excluded if they are scored between 1 and 3 by at least 70% of participants in each stakeholder group and between 7 and 9 by less than 15%. Outcomes not falling into either category will be classified as having no consensus.

### Phase three: online consensus meeting

An invitation will be extended to all Delphi participants to take part in an online consensus meeting. The steering committee will ensure equal representation from each stakeholder group. Meetings will be held to accommodate varying time zones and different participant groups to encourage international participation.

Materials will be circulated prior to the consensus meetings. At each meeting, the results of the RT Delphi will be presented to participants by non-voting facilitators, followed by a discussion of each outcome. Using online polling software, participants will then be invited to anonymously rate each outcome for inclusion in the COS. The options for rating will include 'Yes, critical for inclusion', 'No, important but not critical', and 'Not important for inclusion'. Consensus for an outcome to be included will be based on at least 80% of participants voting 'Yes, critical for inclusion'.

### Ethics and dissemination

Phases two and three of this study were approved by the Royal College of Surgeons in Ireland (RCSI) Research Ethics Committee (202309015).

The results of this study will be presented at national and international scientific meetings and a manuscript will be published in a peer-reviewed journal. A plain English summary will be disseminated and a social media identity will be developed during the early phases of the study. The findings will be published on the RCSI website and shared with postgraduate surgical training programmes nationally and internationally, including through the Intercollegiate Forum. The authors plan to work with RCSI to ultimately integrate the COS into the national higher surgical training programme’s handover curriculum. The authors also aim to present the results to the Health Service Executive Quality and Patient Safety Directorate to assist with national healthcare improvement activities and support future handover policy development. All collaborators will be encouraged to share the findings with relevant national bodies.

## Discussion

Published studies evaluating interventions to improve surgical handover between doctors are clinically and methodologically heterogeneous, making meta-analysis impossible. No COS exists for this area of study, leading to extreme heterogeneity in the types of outcomes measured and reported, and few studies are directly comparable. Over the past 20 years, several organisations have published general guidance on healthcare handover [4–6, 20]; however, none have provided robust evidence-based recommendations for surgical handover, likely due to the quality of the available literature. This protocol describes a multi-stage process to develop the first COS for handover as a step towards supporting higher-quality future research and facilitating better evidence synthesis in this area. Furthermore, it represents the first use of a real-time Delphi survey in a surgical context.

### Trial status

Protocol version 1.0, December 2023.

The quantitative review of phase one is complete and the qualitative review will commence in the near future. Recruitment of participants for phase two has not yet begun. Recruitment is expected to be completed by December 2024.

## Data Availability

The final core outcome set will be submitted for peer review and reported according to the Core Outcome Set-Standards for Reporting (COS-STAR).

## Abbreviations

COMET: Core Outcome Measures in Effectiveness Trials
COS-STAP: Core Outcome Set-Standardised Protocol Items
COS-STAD: Core Outcome Set-Standards for Development
COS-STAR: Core Outcome Set-Standards for Reporting
HCP: Healthcare professional
RT: Real-time
RCT: Randomised controlled trial
